# Development of a Deep Learning System to Detect Esophageal Cancer by Barium Esophagram

**DOI:** 10.3389/fonc.2022.766243

**Published:** 2022-06-21

**Authors:** Peipei Zhang, Yifei She, Junfeng Gao, Zhaoyan Feng, Qinghai Tan, Xiangde Min, Shengzhou Xu

**Affiliations:** ^1^ Department of Radiology, Tongji Hospital, Tongji Medical College, Huazhong University of Science and Technology, Wuhan, China; ^2^ College of Computer Science, South-Central University for Nationalities, Wuhan, China; ^3^ College of Biomedical Engineering, South-Central of University for Nationalities, Wuhan, China; ^4^ Department of Gastroenterology, Tongji Hospital, Tongji Medical College, Huazhong University of Science and Technology, Wuhan, China

**Keywords:** esophageal cancer, barium esophagram, deep learning, image detection, image classification, artificial intelligence

## Abstract

**Background:**

Implementation of deep learning systems (DLSs) for analysis of barium esophagram, a cost-effective diagnostic test for esophageal cancer detection, is expected to reduce the burden to radiologists while ensuring the accuracy of diagnosis.

**Objective:**

To develop an automated DLS to detect esophageal cancer on barium esophagram.

**Methods:**

This was a retrospective study using deep learning for esophageal cancer detection. A two-stage DLS (including a Selection network and a Classification network) was developed. Five datasets based on barium esophagram were used for stepwise training, validation, and testing of the DLS. Datasets 1 and 2 were used to respectively train and test the Selection network, while Datasets 3, 4, and 5 were respectively used to train, validate, and test the Classification network. Finally, a positioning box with a probability value was outputted by the DLS. A region of interest delineated by experienced radiologists was selected as the ground truth to evaluate the detection and classification efficiency of the DLS. Standard machine learning metrics (accuracy, recall, precision, sensitivity, and specificity) were calculated. A comparison with the conventional visual inspection approach was also conducted.

**Results:**

The accuracy, sensitivity, and specificity of our DLS in detecting esophageal cancer were 90.3%, 92.5%, and 88.7%, respectively. With the aid of DLS, the radiologists’ interpretation time was significantly shortened (Reader1, 45.7 s vs. 72.2 s without DLS aid; Reader2, 54.1 s vs. 108.7 s without DLS aid). Respective diagnostic efficiencies for Reader1 with and without DLS aid were 96.8% vs. 89.3% for accuracy, 97.5% vs. 87.5% for sensitivity, 96.2% vs. 90.6% for specificity, and 0.969 vs. 0.890 for AUC. Respective diagnostic efficiencies for Reader2 with and without DLS aid were 95.7% vs. 88.2% for accuracy, 92.5% vs. 77.5% for sensitivity, 98.1% vs. 96.2% for specificity, and 0.953 vs. 0.869 for AUC. Of note, the positioning boxes outputted by the DLS almost overlapped with those manually labeled by the radiologists on Dataset 5.

**Conclusions:**

The proposed two-stage DLS for detecting esophageal cancer on barium esophagram could effectively shorten the interpretation time with an excellent diagnostic performance. It may well assist radiologists in clinical practice to reduce their burden.

## Introduction

Esophageal cancer is the sixth leading cause of cancer-related mortality and the eighth most common cancer worldwide (1). It affects more than 500,000 people globally and its incidence is rapidly increasing (2). Esophagoscopy is the gold standard for esophageal cancer diagnosis, but it is both invasive and expensive (3). Barium esophagram can simultaneously detect morphologic and functional abnormalities in the esophagus and is thus a valuable technique for the assessment of esophageal cancer (4–7). In addition, due to its inexpensive and noninvasive features, as well as its widespread availability, the barium esophagram is usually prioritized over other techniques, such as endoscopy, for clinical diagnostic selection (4). Indeed, in the detection of esophageal malignancy, barium esophagram and endoscopic findings show a good correlation (8). Therefore, barium esophagography represents a cost-effective and useful approach for screening patients with dysphagia (4, 8).

A previous study suggested that barium esophagram is a sensitive modality to diagnose esophageal cancer, and that endoscopy is not routinely recommended to rule out missed tumors in patients who have normal esophagram findings (3). However, inconsistent interobserver interpretations are inevitable among radiologists over diagnosis by conventional visual assessment. In some cases, even experienced radiologists may misinterpret esophagram images and miss esophageal cancer indicators (9). Furthermore, multi-positional esophagography often involves laborious and time-consuming steps that can lead to diagnostic errors. Therefore, in clinical practice, there is a clear need for a method to efficiently interpret a large number of esophagrams, reduce radiologists’ workload, and improve the interpretation accuracy of radiologists with different experience levels.

In recent years, artificial intelligence using deep learning algorithms has made remarkable progress in medical imaging. Researchers have used deep learning to improve the diagnosis of various gastrointestinal cancers and precursor lesions, such as esophageal cancer, gastric cancer, and colorectal neoplasm (10–23). Gehrung et al. (10) used a deep learning framework to analyze samples of Cytosponge-TFF3 test, a minimally invasive alternative to endoscopy, for detecting Barrett’s esophagus, a precursor of esophageal adenocarcinoma. Guo et al. (11) developed a deep learning model for real-time diagnosis of precancerous lesions and early esophageal cancer both in endoscopic images and video settings. However, most of these studies are based on endoscopy or histopathology, and no reports have so far assessed the usefulness of deep learning in the diagnosis of esophageal cancer based on barium esophagrams. To the best of our knowledge, the study by Yang et al. (9) in 2017 is the only report focused on the computer-aided diagnosis of esophageal barium meal; however, the researchers used traditional machine learning algorithms (SVM and KNN), and the extracted features were limited. In 2019, Togo et al. (24) developed a deep learning model based on gastric barium images to investigate automated gastritis detection, reporting a sensitivity of 0.962 and a specificity of 0.983. Their study suggested that deep learning techniques can live up to expectations and perform well in barium meal examinations. Therefore, in view of the wide application and important clinical value of the barium esophagram, we used a deep learning approach for detecting esophageal cancer using a large number of barium esophagram images.

In this study, we developed an automated DLS to detect esophageal cancer on barium esophagram. The two-stage deep learning architecture applied in our study is a major advantage because it makes full use of the effective esophagography features so as to improve the final diagnostic accuracy. Additionally, we compare the diagnostic efficacy of our DLS with that of radiologists and three popular detection networks, namely YOLO (You Only Look Once) (25), MobileNetV3 (26), and EfficientnetV2 (27).

## Methods

This study was approved by the Institutional Review Board of our hospital. Written informed consent was waived for this retrospective anonymized study.

### Overview of the Algorithm Framework

**Figure 1:**
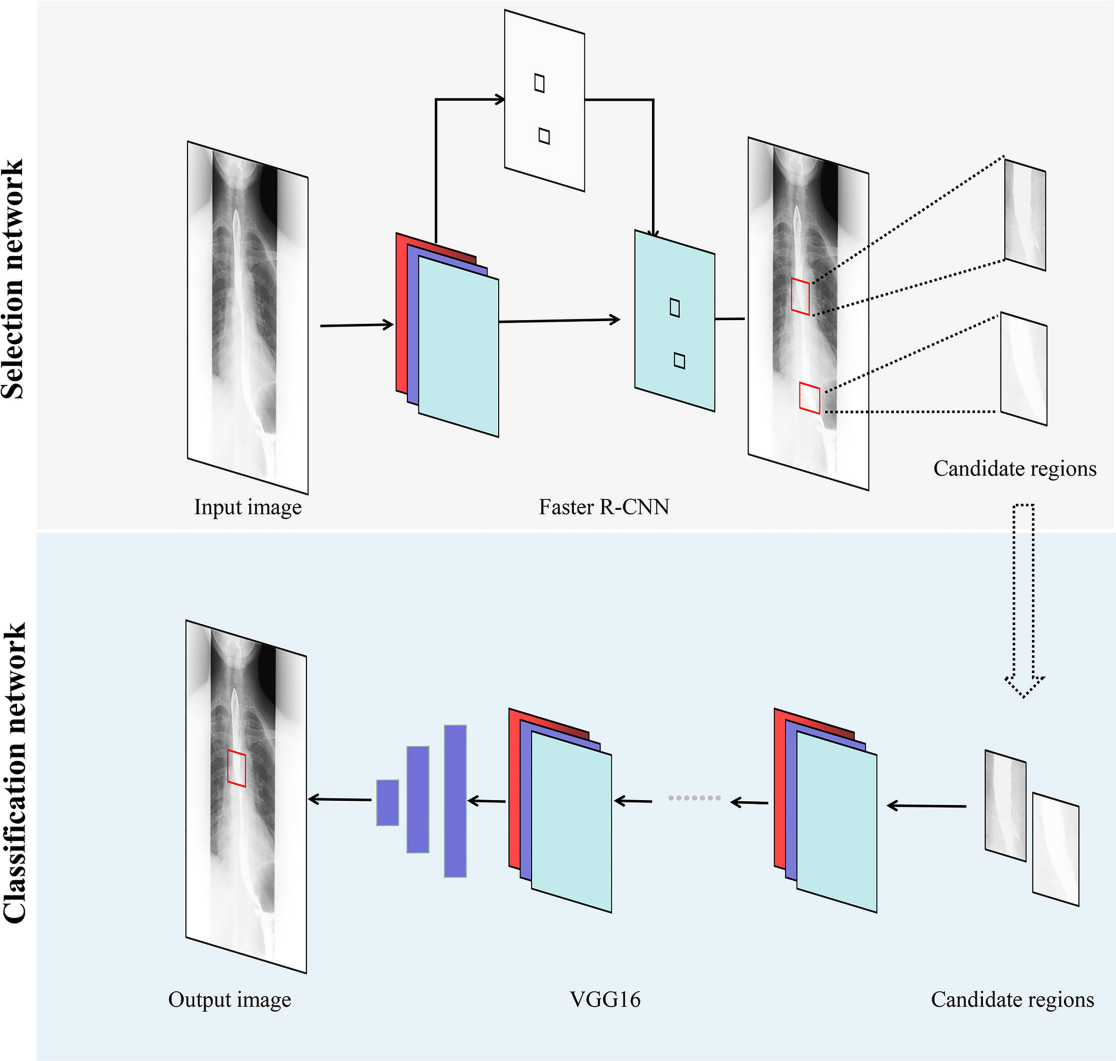
Overview of our two-stage DLS for esophageal cancer detection. Faster R-CNN, faster region-based convolutional neural network; VGG, visual geometry group; DLS, deep learning system.

A schematic diagram of the proposed method is shown in **Figure 1**. In this study, we designed a DLS with two network models: a Selection network, used to identify the candidate regions, and a Classification network, employed to classify the proposed regions. The selected candidate regions from the Selection network were defined as the input of the Classification network. Finally, a test set was used for assessing the efficiency of the two-stage DLS (combination of the Selection network and the Classification network).

### Datasets

Images were retrospectively collected from patients who underwent barium esophagography in our hospital from January 2017 to June 2019. Positive and negative groups were set based on clinical, radiographic, endoscopic, and surgical findings. Before study initiation, cases with unmatched results between the radiographic report and esophagoscopy or pathology, as well as those with poor image quality, were excluded. All barium examinations of the patients with dysphagia were completed by gastrointestinal radiologists. X-ray images taken at multiple positions were used for a comprehensive evaluation of the condition of the esophagus. In the vast majority of patients, these images were obtained at: 1) the anteroposterior view; 2) the right anterior oblique view; and 3) the left anterior oblique view. All data were stored in Digital Imaging and Communications in Medicine (DICOM) format. All included cases of esophageal cancer were confirmed pathologically. In total, 6445 images were obtained from 200 patients with esophageal cancer, and 11,352 images were obtained from 299 patients without esophageal cancer. All images of esophageal cancer lesions were annotated by a board-certified radiologist. Using LabelImage software (GitHub, Inc., San Francisco, CA, USA), a rectangular bounding box was drawn on the barium esophagram for esophageal cancer detection. During the annotation process, the results of the radiographic report, esophagoscopy, or surgical pathology were referred to side-by-side. The labeled images were then reviewed by another radiologist with 12 years of clinical experience.

Five datasets were used for stepwise training, validation, and testing of the DLS: 1) 5279 images with annotated esophageal cancer lesions from 160 patients were retrieved as training set for the Selection network; 2) 1166 images with esophageal cancer lesions from 40 patients were retrieved as testing set for the Selection network; 3) 4611 cancer/7815 no-cancer images derived from 112 cancer/182 no-cancer patients, respectively, were used to train the Classification network; 4) 668 cancer/1990 no-cancer images from 48 cancer/64 no-cancer patients were used to validate the efficiency of the Classification network; 5) 1166 cancer/1547 no-cancer images from 40 cancer/53 no-cancer patients were used to test the Classification network. Dataset 5 was also interpreted by two radiologists, with and without the aid of our DLS, to evaluate the accuracy of the latter in detecting esophageal cancer.

### Preprocessing

Images in the datasets were preprocessed before the construction of the deep learning networks. The images were subjected to image augmentation techniques such as horizontal flipping, cropping, and random rotation, and normalized by dividing each pixel value by 255 before being input into the networks.

### Development of the Algorithm

Two deep learning networks were used to develop our two-stage DLS. The DLS was implemented with TensorFlow as the backend, on a desktop computer equipped with a Linux operating system, 64 GB RAM, and a single GeForce RTX 2080 Ti GPU (Nvidia, Santa Clara, CA, USA).

First, to detect suspected esophageal cancer regions, we designed the Selection network based on the faster region-based convolutional neural network (Faster R-CNN). The Selection network was developed to extract relevant regions related to esophageal cancer and discard irrelevant ones. In the Selection network, barium esophagram images are input to the backbone network to extract features. Region proposal network generates region proposals by these features. For region proposals of different sizes, ROI pooling layer maps their feature vectors to the same size. Finally, the feature vectors of these region proposals are input into the Fast R-CNN predictor network to output the probability and location of suspected esophageal cancer regions. The Keras API (https://keras.io/) with TensorFlow (https://www.tensorflow.org/) backend was used for the implementation of the Faster R-CNN. While training the Faster R-CNN, only images with esophageal cancer lesions were used to develop the Selection network. Datasets 1 and 2 were retrieved as training and validation sets, respectively.

Then, to refine the classification results and reduce false positives, the Classification network was developed to classify the candidate regions extracted in the Selection network. The Classification network is mainly composed of convolutional layers, which extract features from candidate regions, and linear layers, into which these features are input to derive classification results. The Classification network outputted the probability of whether the detection result obtained from the Selection network was esophageal cancer or no esophageal cancer. Datasets 3 and 4, described in the previous subsection, were used for training and validating the Classification network, which was derived from the Visual Geometry Group 16 (VGG16) convolutional neural network. During the development of the Classification network, all the images were first fed into the Selection network, and the proposed candidate regions were then fed into the Classification network.

We utilized the ImageNet to pretrain the backbone subnetwork in the Selection Network. For other subnetworks in the Selection network, we used truncated normal distribution (mean=0.0, stddev=0.001) to initialize network parameters. We fine-tuned the parameters with the images of barium esophagram included in the current study. The momentum method was used to optimize the parameters of the Selection network. The initial learning rate and momentum coefficient in the momentum optimizer were set as 0.001 and 0.9, respectively. The batch size was set at a value of 1 and the intimal iterations were set as 70000. Similarly, we utilized the ImageNet to pretrain the Classification network. We fine-tuned the parameters with the regions of interest included in the current study. The Adam was used to optimize the parameters of the Classification network. The initial learning rate was set as 0.001. The batch size was set at a value of 32 and the intimal epochs were set as 50.

**Figure 2:**
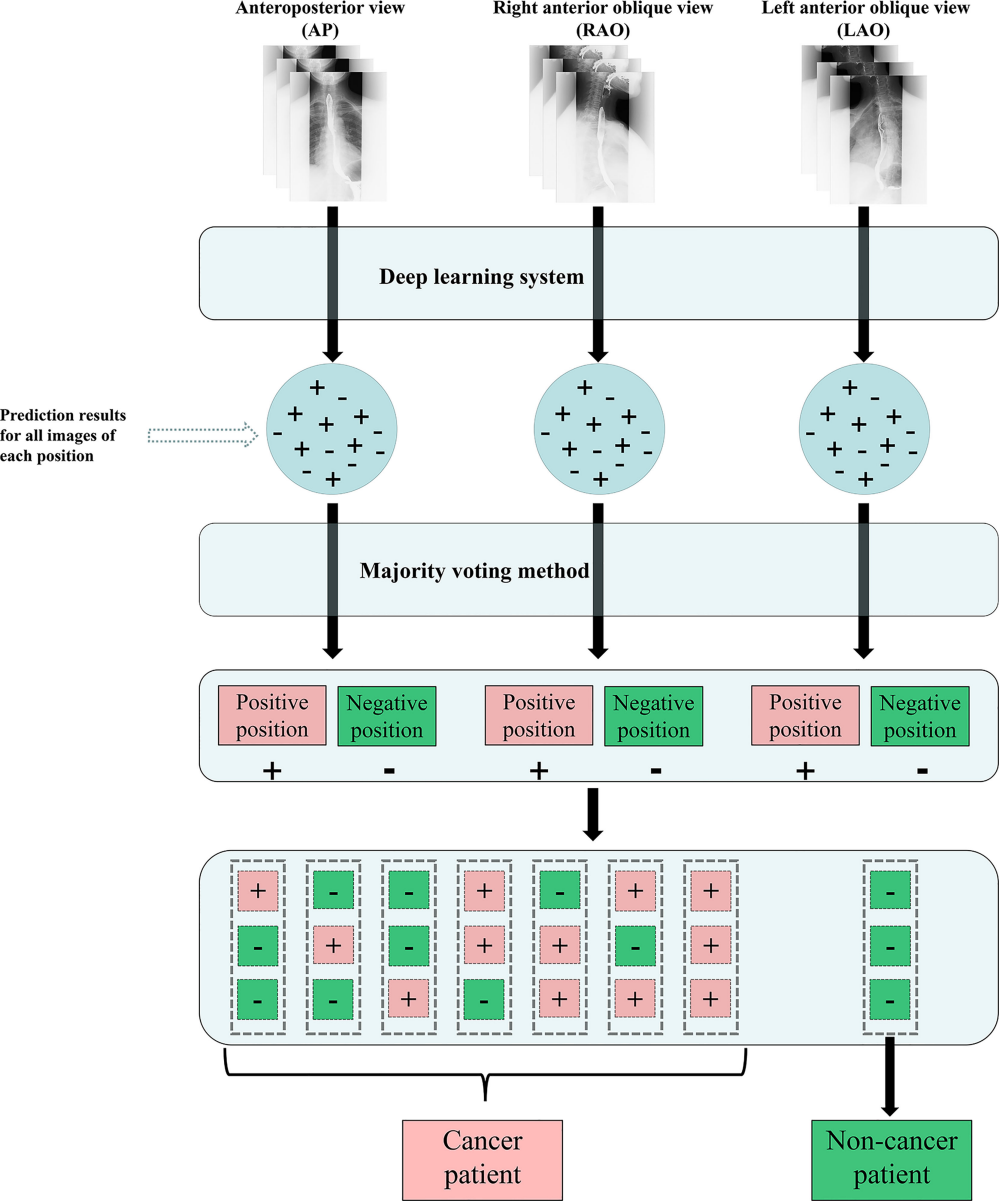
Flow chart of the DLS approach for esophagram-based esophageal cancer diagnosis. (+) esophageal cancer; (-) no esophageal cancer; DLS, deep learning system.

To mimic the real clinical interpretation of images by radiologists, for the evaluation of the testing data (Dataset 5), a majority voting method was applied to integrate estimated results of images acquired in each position. For patients in the testing data, a patient was defined as a cancer patient if at least one position was predicted as esophageal cancer; otherwise, the patient was defined as a no-cancer patient. Once a position was predicted as esophageal cancer, the prediction location integrated by majority voting was selected as the cancer location (see **Figure 2**).

### Evaluation of the Algorithm

First, we used Dataset 2 to test recall, precision, and average precision (AP) of the Selection network and compared these metrics with those of the YOLO network (25). Our Selection network was based on the Faster R-CNN architecture, a two-stage detection method, while YOLO is a single-stage object detection method. Second, we used Dataset 5 to test the accuracy, sensitivity, specificity, and AUC of our two-stage DLS and compared these metrics with two other networks, MobileNetV3 (26) and EfficientnetV2 (27). MobileNetV3 is a relatively popular lightweight network, while EfficientV2 is a popular high-performance network. If the Intersection over Union (IoU) between the detection result of the classification dataset and annotation data was more than 0.3, the detection result was defined as a true positive; otherwise, it was defined as a false positive.

Dataset 5 was assigned to two radiologists (with 6 and 4 years of clinical experience) for random interpretation either with or without the aid of the algorithm. Following a 2-week washout period, the same selected sample was randomly interpreted again by the same corresponding radiologist without or with aid of the algorithm in a crossover design (i.e., if the first read was with algorithm aid, then the second read was without algorithm aid, and vice versa). Patient classification, cancer location, and time to diagnosis were recorded for each interpreted position.

### Statistical Analysis

We evaluated the efficacy of the Selection network using the precision-recall (PR) curve to assess AP in Dataset 2. Receiver operating characteristic (ROC) analysis was performed to evaluate the classification performance of the DLS. The sensitivity and specificity for both image-based analysis and case-based analysis of the DLS on the testing dataset were calculated. In addition, ROC curves were also generated for the detection results of two radiologists with or without DLS aid. The paired sample t-test was used to assess the difference in the interpretation time for evaluating each case. P-values of less than 0.05 were considered statistically significant. R (version 3.6.0, available at http://www.R-project.org/) was used for statistical analyses.

## Results

The AP, precision, and recall of the Selection network on the testing dataset were 70.6%, 74.7%, and 91.7%, respectively, suggesting that 1069 of the 1166 positive lesions can be identified. In turn, the AP, precision, and recall of the YOLO network on the testing dataset were 60.8%, 64.1%, and 82.9%, respectively. On case-based analysis, the accuracy, sensitivity, specificity, and AUC of our DLS on the testing dataset were 83.7%, 92.5%, 88.7%, and 0.906, respectively. This means that 37 of the 40 esophageal cancer patients could be identified. On case-based analysis, the accuracy, sensitivity, specificity, and AUC of MobileNetV3 on the testing dataset were 80.5%, 81.6%, 78.6%, and 0.891, respectively. On case-based analysis, the accuracy, sensitivity, specificity, and AUC of EfficientNetV2 on the testing dataset were 81.2%, 87.8%, 70.0%, and 0.881, respectively.

**Figure 3:**
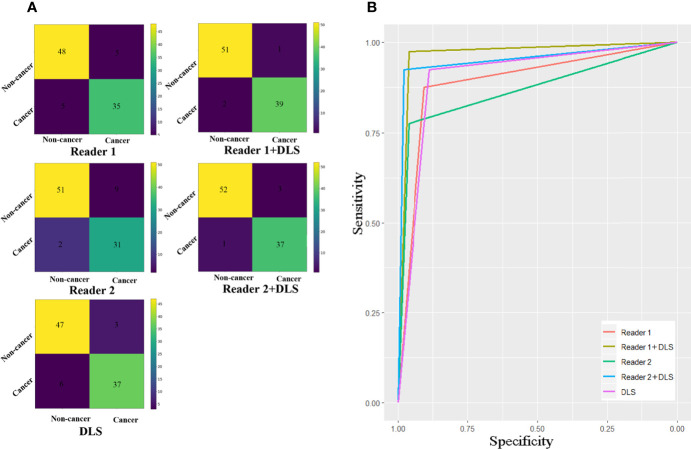
Confusion matrixes **(A)** and ROC curves **(B)** for the five classification methods. The AUC of Reader1+DLS and Reader2+DLS are significantly higher than the AUC of Reader1, Reader2, and DLS (all *P* < 0.05). DLS, deep learning system; ROC, receiver operating characteristic; AUC, area under the ROC curve.

Classification results of the testing dataset for the five classification methods are presented in **Table 1** and **Figure 3**. For Reader1, case-based AUC with and without DLS aid was 0.969 and 0.890, respectively. For Reader1 without DLS aid, the accuracy, sensitivity, and specificity for detecting esophageal cancer were 89.3%, 87.5%, and 90.6%, respectively. For Reader2, case-based AUC with and without DLS aid was 0.953 and 0.869, respectively. For Reader2 without DLS aid, the accuracy, sensitivity, and specificity for detecting esophageal cancer were 88.2%, 77.5%, and 96.2%, respectively (**Table 2**). The classification efficiency of the combination of radiologists’ conventional visual inspection approach and DLS was significantly higher than that of the two methods alone (*P*<0.05). Thus, for both radiologists, diagnostic efficiency was significantly improved by DLS (**Figure 3**).

**Table 1:** Classification results for the testing dataset.

		DLS		Reader1		Reader1+DLS		Reader2		Reader2+DLS	
		(+)	(–)	(+)	(-)	(+)	(-)	(+)	(-)	(+)	(-)
True label	(+)	37	3	35	5	39	1	31	9	37	3
	(-)	6	47	5	48	2	51	2	51	1	52

(+) esophageal cancer; (-) no-esophageal cancer; DLS, deep learning system.

**Table 2:** Comparison of esophageal cancer detection performance for the five classification methods.

	Time (Seconds)	AUC	Sensitivity (%)	Specificity (%)	Accuracy(%)
DLS	*NA*	0.906	92.5	88.7	83.7
Reader1	72.2	0.890	87.5	90.6	89.3
Reader1+DLS	45.8	0.969	97.5	96.2	96.8
Reader2	108.7	0.869	77.5	96.2	88.2
Reader2+DLS	54.1	0.953	92.5	98.1	95.7

DLS, deep learning system; NA, not applicable.

**Figure 4:**
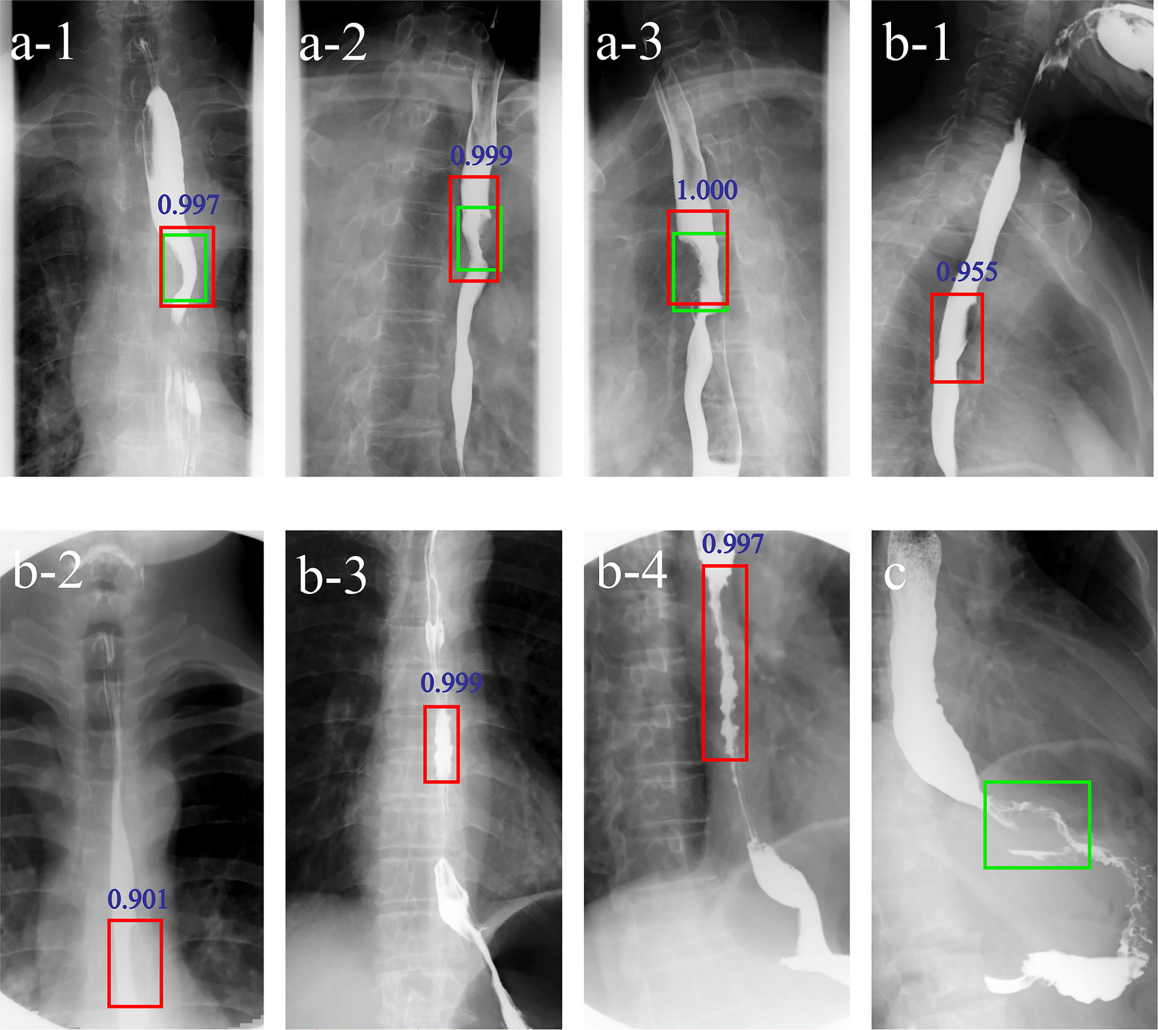
Examples of DLS-diagnosed images. The green boxes indicate the ground truth annotated by radiologists. The red boxes and blue numbers indicate the positioning boxes and probability values outputted by the DLS. **A**-**1-A**-**3** are true positive cases. **B**-**1-B**-**4** are false positive cases. **(C)** is a false negative case. **A-1**-**3** represent three positions (anteroposterior, right anterior oblique, and left anterior oblique view) of barium esophagrams from a 71-year-old male esophageal cancer patient. A filling defect and eccentric stenosis in the middle of the esophagus can be noticed. **B-1**, **B**-**2** represent misdiagnosis of esophageal cancer based on the false filling defect caused by gas. **B**-**3**, **B**-**4** represent misdiagnosis of esophageal cancer based on tertiary waves (red boxes) in a 64- and a 78-year-old patient, respectively. **(C)** represents missed diagnosis of esophagogastric junction cancer. *DLS*, deep learning system.

Applying the DLS alone, three false negative and six false positive cases were detected. For Reader1, with DLS aid 4/5 false negative cases were correctly classified into the positive group and 5/5 false positive cases were correctly classified into the negative group. For Reader2, with DLS aid 6/9 false negative cases were correctly classified into the positive group and 2/2 false positive cases were correctly classified into the negative group. **Figure 4** shows examples of false negative and false positive barium esophagrams.

**Figure 5:**
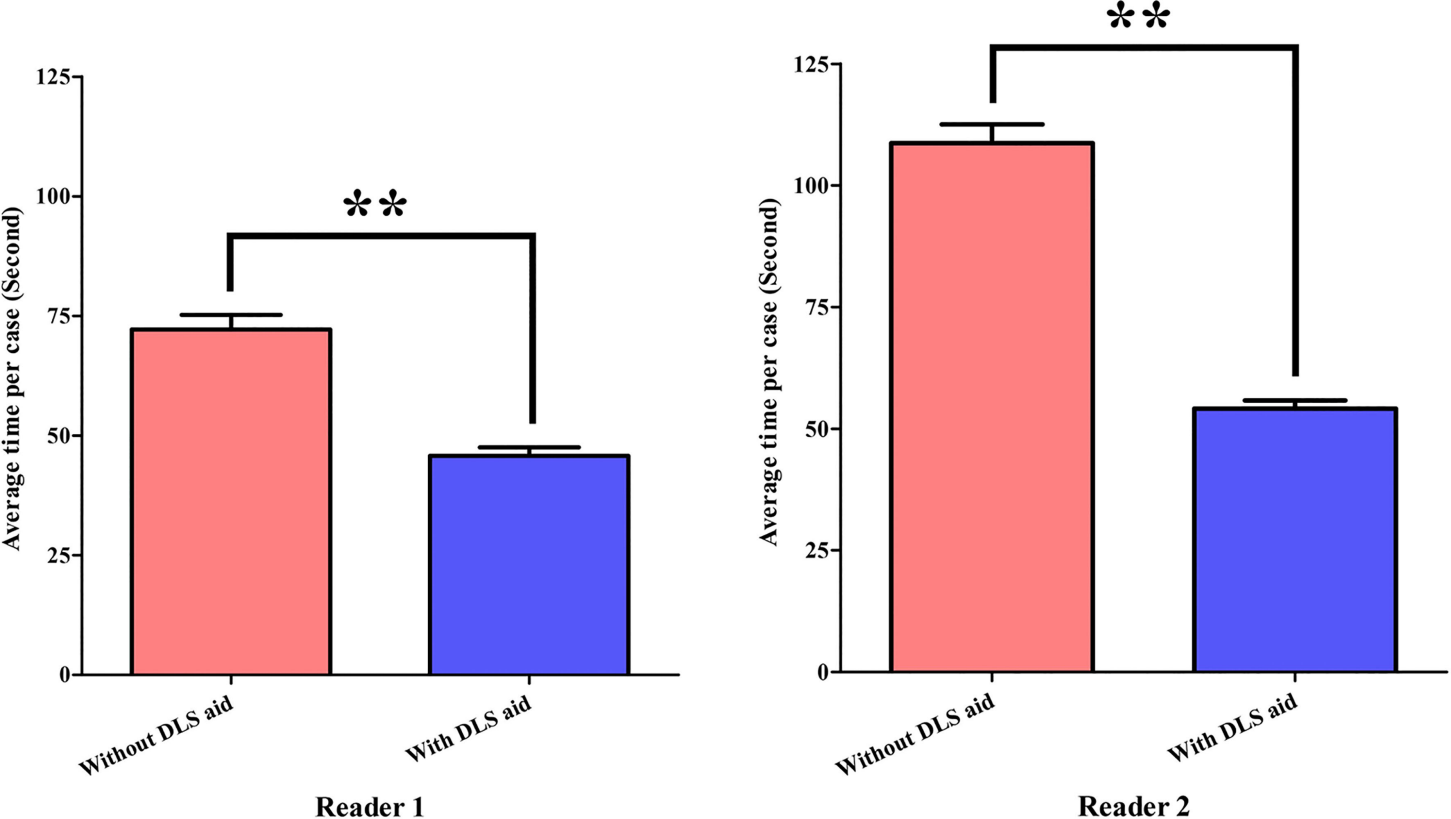
Statistical comparison of radiologists’ interpretation time. ^**^ *P*<0.01. DLS, deep learning system.

The interpretation time of Reader1 without and with DLS aid took on average 72.2 s and 45.8 s, respectively. For Reader2, interpretation time without and with DLS aid took 108.7 s and 54.1 s, respectively. With the aid of DLS, the interpretation time was significantly shortened for both radiologists (both *P*<0.01) (**Figure 5**).

## Discussion

In this study, we show that DLS-based automatic detection of esophageal cancer on barium esophagram can significantly aid radiologists’ diagnosing task by relieving the burden of a time-consuming image review process and minimizing the heterogeneity caused by variable experiences of radiologists in clinical practice. Additionally, the proposed DLS can ensure the robustness and effectiveness of fully automated detection and location of cancerous foci through decoupling the identification and classification features. Our results indicate that deep learning has the potential to become an important add-on for radiologists facing a large number of barium esophagram images by facilitating esophageal cancer screening. The present study further suggests that DLS can reduce unnecessary endoscopy for negative patients so as to save labor and resources. To the best of our knowledge, this is the first report that evaluates the ability of a CNN model to detect esophageal cancer in barium esophagrams.

Deep learning has been applied for the detection of esophageal cancer mostly based on endoscopy or pathological images. Only a few studies applied it to barium examination. Togo et al. (24) developed a DCNN-based automated gastritis detection system by using 6520 gastric barium images from 815 subjects. Sensitivity and specificity of this study were 0.962 and 0.983, respectively. The authors claimed that deep learning techniques may be effective for differentiating gastritis and non-gastritis on gastric barium images. In addition, Yang et al. (9) proposed a CAD system for diagnosing esophageal cancer by using 300 original esophageal X-ray images and found that the classification performance of the SVM and K-nearest neighbors outperformed the conventional visual inspection approaches in terms of diagnostic quality and processing time. However, in contrast to our study, those reports provided neither a positioning box nor evaluated the added value to radiologists.

Our DLS can accurately identify and locate esophageal cancer on barium esophagram and provide a positioning box with a probability value. A comparison of the diagnostic value of radiologists’ conventional visual inspection approach and DLS showed higher sensitivity and AUC for the DLS method. In turn, our study indicated that the classification efficiency of combined conventional visual inspection and DLS is significantly higher than that of the two methods alone. Thus, diagnostic efficiency is significantly improved by the implementation of the proposed DLS. Moreover, interpretation time is significantly shortened for radiologists when aided by the DLS. In line with previous studies (28–34), our results further confirm the potential of deep learning in medical image classification and detection through the ability of deep learning networks to extract and analyze massive data features.

The two-stage deep learning architecture of the model proposed in our study is a major advantage, in that it makes full use of the effective esophagram features so as to improve the final diagnostic accuracy. In the first step, the Selection network extracted important regions related to esophageal cancer as candidate regions. Next, the Classification network classified these candidate regions into positive and negative groups. As a consequence, interference from irrelevant information was reduced in the Classification network. Comparative experiments showed that the recall, precision, and AP of our Selection network are higher than those computed using YOLO, while the accuracy, sensitivity, specification, and AUC of our Classification network are higher than those of MobileNetV3 and EfficientNetV2. Nevertheless, some false positive and false negative cases were also observed in our DLS. The prediction results showed that the tertiary waves are a major factor leading to misdiagnosis in the proposed DLS. Tertiary waves are characterized by intermittently weakened or absent peristalsis associated with multiple nonperistaltic contractions of varying severity (4, 35), whose incidence increases in older patients and in many primary or secondary esophageal motility disorders (36, 37).

Distinguishing tertiary waves from esophageal cancer is difficult for DLS but easy for experienced radiologists. Benign esophageal strictures typically appear as relatively symmetric segments of narrowing with smooth contours and tapered margins, whereas malignant structures are more asymmetric and have nodular, irregular, or ulcerated contours and abrupt, shelflike margins (4). Other reasons, such as the inability of the entire esophageal circumferential sheath to contract freely, or delayed bolus clearance due to adhesions to the aorta or mediastinal lymph nodes, can also be responsible for the false positive/negative classification (36). Additionally, the radiologic identification of some cancers at the distal esophagus and esophagogastric junction remains a challenge to the deep learning detection system. In general, the conventional visual inspection approach and the DLS are complementary in the detection of esophageal cancer, and their combination can achieve an almost perfect diagnostic performance. In this regard, the higher sensitivity of the DLS greatly helps in meeting the clinical need to minimize false negatives. These results are important to consider in terms of daily practice where false positives are generally more acceptable than false negatives. To improve this system, we need to select training images that include such cases more often.

Many gastroenterologists traditionally believe that endoscopy is required to detect esophageal cancer possibly missed on barium esophagography (4). However, studies showed that the latter can detect esophageal cancer with a sensitivity greater than 95% (3, 8, 38). In addition, only about 1% of patients who underwent barium esophagography are recommended to receive endoscopy for malignancy exclusion (3). Some gastroenterologists advocate in turn the use of endoscopy and biopsy to exclude esophageal cancer for all patients with radiographically diagnosed esophageal strictures, because it is relatively difficult to identify benign strictures from circumferentially infiltrating cancers on barium esophagram (4). Nevertheless, a study reported that no malignant tumors were identified through endoscopic biopsy in subjects who were diagnosed with unequivocally benign-appearing strictures on barium esophagram (39). Therefore, endoscopy may not be necessary to rule out esophageal cancer in these patients. Along these lines, the present study also supports the notion that endoscopy is not required for patients with negative esophageal cancer screening results on barium examination.

In summary, we designed a DLS for automatized and accurate detection of esophageal cancer based on barium esophagram. The usefulness of our model in clinical practice can be further anticipated in light of the diagnostic effectiveness of X-ray imaging-based deep learning techniques demonstrated by previous studies (40–42). Admittedly, our study has some inevitable limitations. First, all data were collected from a single medical center, hence there was a potential for selection bias. Further multi-center studies are thus warranted to confirm the reliability of our prediction system. Second, the image sequences used in our study were not always consistent. Due to the case-based tailored and flexible approach to image acquisition for different medical scenarios, variability existed for the position and number of images for each patient. In future studies, image standardization should be highly considered. Third, we used only high-quality barium esophagram images for the training and test images. Thus, we are not sure if the DLS can effectively diagnose esophageal cancer using low-quality images, such as those with halation, mucus, blur, or that are out of focus. Therefore, we will further verify the generalization ability of our system while normalizing the images in the future.

## Conclusions

To date, no research had been conducted on the detection of esophageal cancer on barium esophagography using deep learning methods. Our study showed that a DLS involving a two-stage model can achieve a positioning box with a probability value, significantly shorten interpretation time, and improve the sensitivity and accuracy of esophageal cancer detection by radiologists.

## Data Availability Statement

The original contributions presented in the study are included in the article/supplementary material. Further inquiries can be directed to the corresponding authors.

## Ethics Statement

The studies involving human participants were reviewed and approved by Tongji Hospital, Tongji Medical College, Huazhong University of Science & Technology. The ethics committee waived the requirement of written informed consent for participation.

## Author Contributions

XM and SX contributed to the study conception and design. Material preparation and data collection were performed by PZ, ZF, and QT. Data analysis and interpretation: YS, PZ, XM, and SX. The first draft of the manuscript was written by PZ, YS, and JG. All authors commented on previous versions of the manuscript. All authors read and approved the final manuscript.

## Funding

This research was supported by the National Natural Science Foundation of China Grant No. 81801668, 61773408, the Natural Science Foundation of Hubei Province Grant No. 2020CFB541, and the Fundamental Research Funds for the Central Universities, South-Central MinZu University (CZY22015).

## Conflict of Interest

The authors declare that the research was conducted in the absence of any commercial or financial relationships that could be construed as a potential conflict of interest.

## Publisher’s Note

All claims expressed in this article are solely those of the authors and do not necessarily represent those of their affiliated organizations, or those of the publisher, the editors and the reviewers. Any product that may be evaluated in this article, or claim that may be made by its manufacturer, is not guaranteed or endorsed by the publisher.
